# Strongyloides Hyperinfection Presenting as a Gastric Outlet Obstruction

**DOI:** 10.7759/cureus.6603

**Published:** 2020-01-08

**Authors:** Fariba Yazdanpanah, Helena Saba, Rabin Rahmani, Z. Jacob Schreiber, Pierre Hindy

**Affiliations:** 1 Gastroenterology, Gastroenterology Associates of Brooklyn, Brooklyn, USA; 2 Gastroenterology, Maimonides Medical Center, Brooklyn, USA; 3 Pathology, Gastroenterology Associates of Brooklyn, Brooklyn, USA

**Keywords:** gastric outlet obstruction, strongyloidiasis, strongyloides, strongyloides, autoinfection, hyperinfection

## Abstract

Strongyloides is a unique nematode in its ability to cause a secondary hyperinfection and disseminated disease several years following initial contact. The prevalence of Strongyloides infection has been rising; it is currently considered a global disease, which presents with a broad spectrum of clinical signs and symptoms among patients. This case report focuses on a 67-year-old Caribbean female presenting with severe weight loss, vomiting, early satiety, and mild anemia who was subsequently diagnosed with strongyloidiasis on the basis of a duodenal biopsy pathology report obtained via esophagogastroduodenoscopy (EGD).

## Introduction

The history of Strongyloides’ discovery goes back to 1876 when Dr. Louis Normand made a revelatory observation in Toulon, France [1]. He reported the presence of a worm (of about 0.25 mm in length) in the feces of French soldiers suffering from profuse diarrhea after repatriating from Cochinchina (Vietnam).

Three years later, in 1879, Grassi and Parona, two reputable physicians in Italy, called this parasite "Strongyloides" (the Greek terms στρονγύλος meaning "round" and εϊδος meaning "similar") [1].

The Strongyloides family is comprised of at least 50 species but S. stercoralis is the most prevalent in humans [2-3]. Although this parasite is more commonly found in subtropical and tropical regions, some studies have pointed out the significance of S. stercoralis-associated disease as a recently emerging global issue that has been reported in developed countries, more particularly, in the United States and the United Kingdom, especially among immigrants and travelers returning from endemic areas [4-6].

The complications associated with Strongyloides infection rely on its complex life cycle. In fact, the parasite has the ability to infect humans and replicate within a host body (autoinfection) right before entering a latency phase which could last years or even decades. To note, most patients with positive serology for S. stercoralis are asymptomatic. However, in the case where an originally immunocompetent host experiences any form of weakening of his/her immune system (whether due to the use of immunosuppressive medications, infection with human immunodeficiency virus (HIV) or the human T-cell leukemia virus, type 1 (HTLV-1), or even the worsening severity of chronic diseases, etc.), the S. stercoralis nematode will systematically multiply in an uncontrollable fashion (hyperinfection) [4-7] and will likely disseminate its larvae to several internal organs, thus resulting in a life-threatening condition. 

## Case presentation

Our patient is a 67-year-old Jamaican female who presented with a 76 lb weight loss over the span of a year. She had lost her appetite and had multiple episodes of vomiting. There was no history of fever, cough, abdominal pain, or diarrhea. She had been recently diagnosed with new-onset diabetes mellitus.

On physical exam, the patient was not in distress, although she appeared fatigued, showed some signs of dehydration, and was cachectic with a body mass index (BMI) of 17. Her vitals demonstrated low blood pressure. The remainder of the physical exam was unremarkable. In view of her symptoms and presentation, she was transferred to the nearby hospital for further care and treatment.

Laboratory workup revealed mild normocytic normochromic anemia with a hemoglobin of 11.7 g/dL (n = 12.0 - 16.0 g/dL), white blood cell count within the normal range (including an eosinophil count of 2.7 (n = 0.3 - 5.9%)), a high platelet count of 456 K/uL (n = 150 - 400 K/uL), hyponatremia, and hypoalbuminemia. Other electrolytes and liver and kidney function were unremarkable. Additional workup showed a hemoglobin (Hb) A1c of 5.8 and reactive HTLV-I-II antibodies.

**Figure 1:**
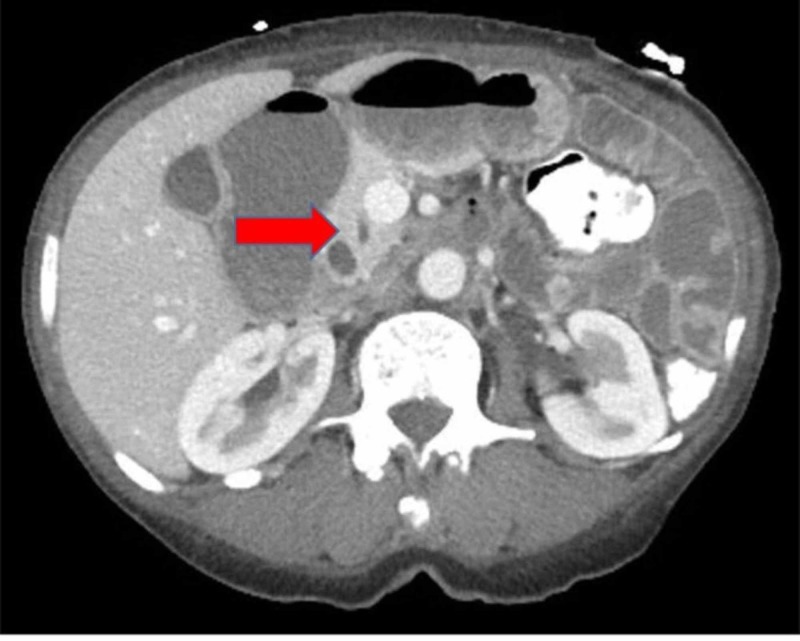
A computed tomography (CT) image with contrast demonstrates a gastric outlet obstruction, along with tapering of the third portion of the duodenum

An abdominal computed tomography (CT) revealed a picture of gastric outlet obstruction consistent with proximal dilatation of the duodenum and stomach, along with dilation of the second portion of the duodenum and tapering of the third portion (Figure 1).

**Figure 2:**
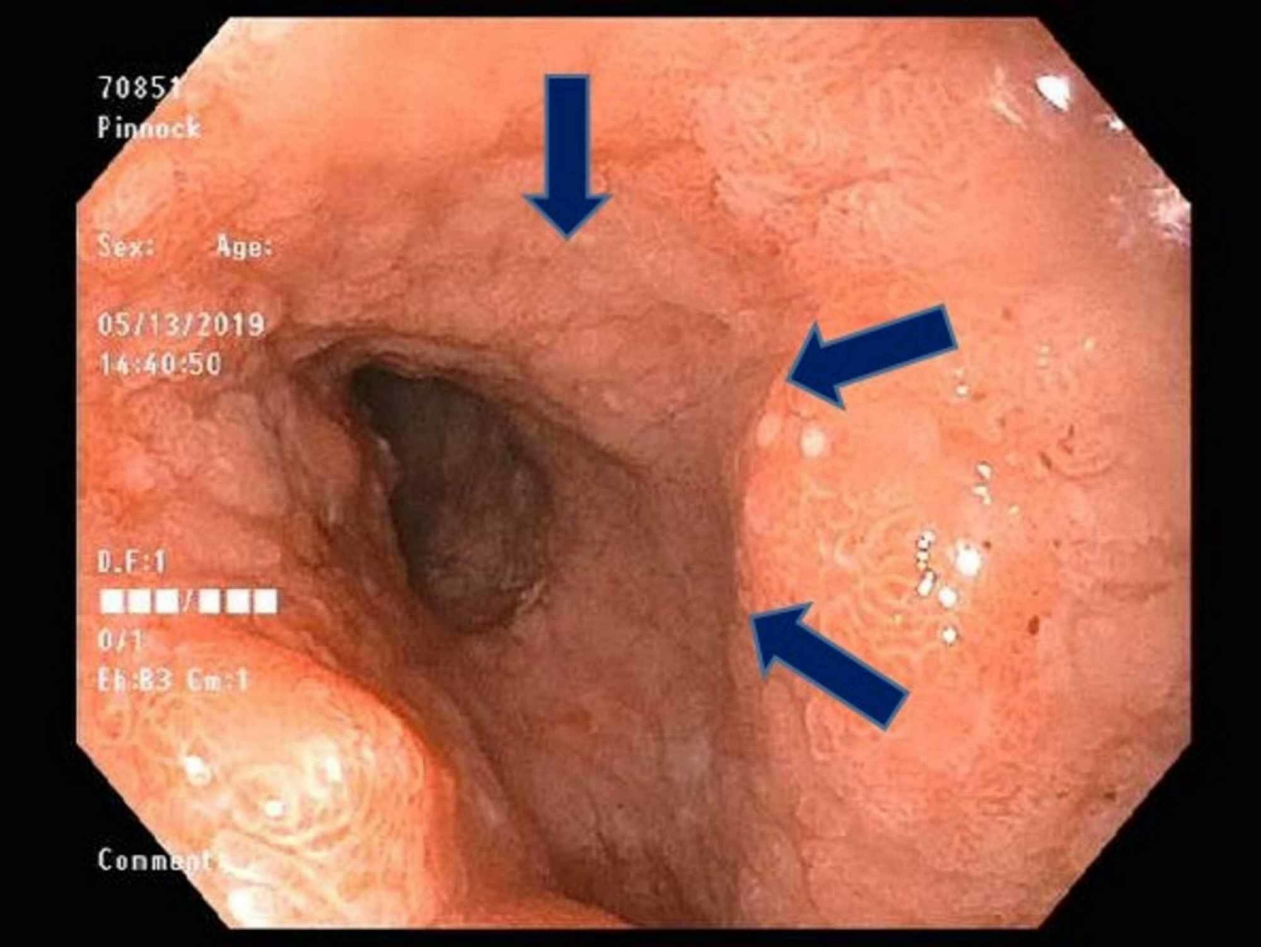
Endoscopic view of narrowing in the distal part of the duodenum

Subsequently, an upper endoscopy was performed and revealed a narrowing in the third portion of the duodenum with ulcerated erythematous and congested mucosa in the proximal duodenum (Figure 2).

**Figure 3:**
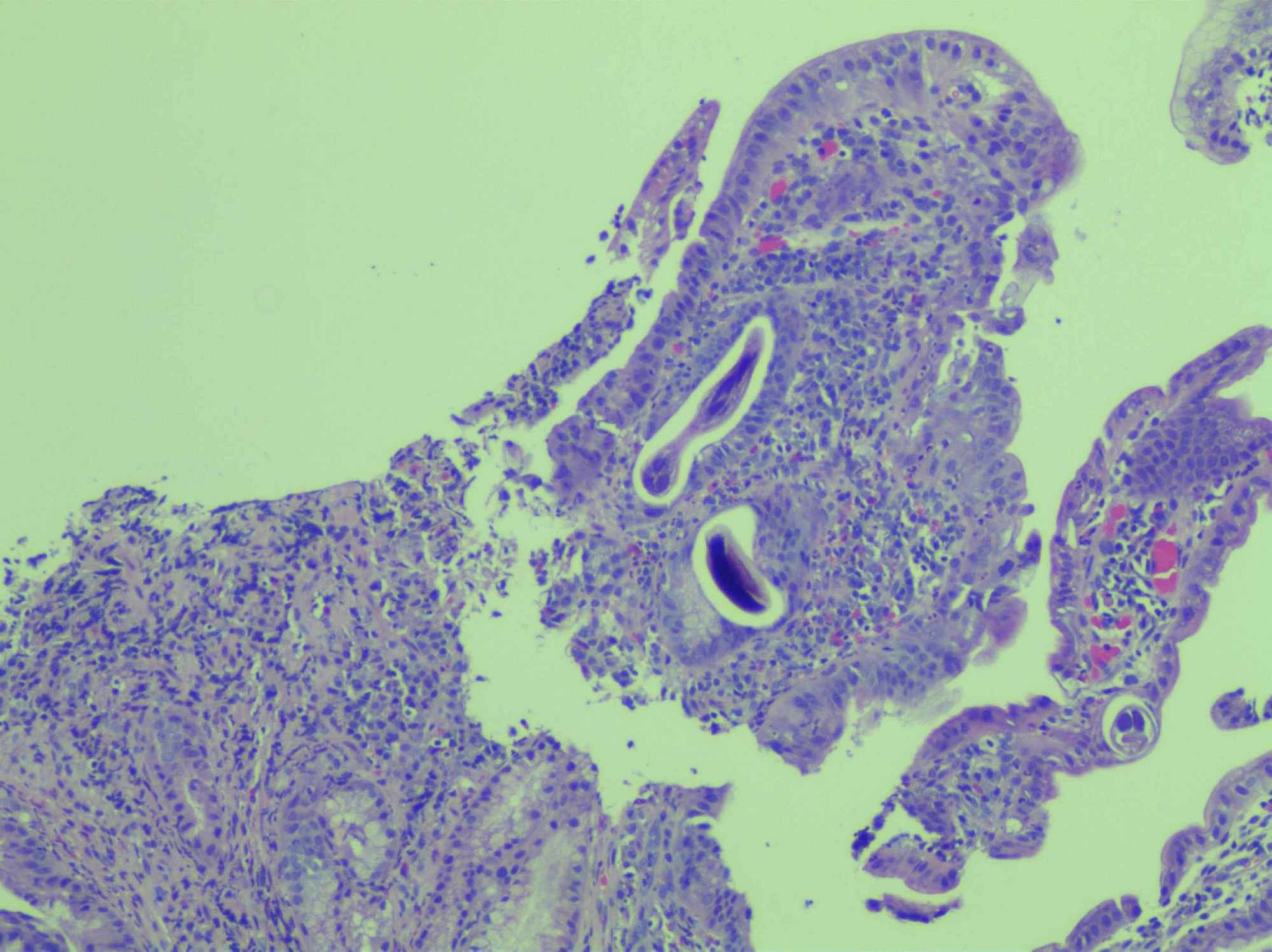
Histology shows acute and chronic inflammation of the duodenal mucosa with a cross-section of parasitic worms, consistent with Strongyloides stercoralis

Biopsies taken from the duodenum revealed the presence of acute inflammation and parasite fragments in the duodenal mucosa consistent with an S. stercoralis infection (Figure 3).

Once the diagnosis of Strongyloides was established, ivermectin, at a recommended dose for her weight, was given. Within a few days, she started feeling much better and was subsequently discharged. Her appetite clearly improved and her vomiting subsided. After two weeks, she gained 8 lb (3.6 kg), as assessed on a follow-up visit. Stool examination post-treatment did not reveal the presence of any parasites.

## Discussion

S. stercoralis is a nematode with a complex life cycle. The parasite infects humans and replicates within its host for years or even decades (autoinfection). Even with positive serologies for S. stercoralis, patients are usually asymptomatic.

Immunosuppressive medications, HIV, HTLV-I and II, chronic diseases, among other etiologies, allow successful and unlimited replication of the parasite with subsequent life-threatening dissemination of its larvae to multiple internal organs; this phenomenon is called hyperinfection.

HTLV infection interferes with the body’s natural ability to mount an immune response against Strongyloides spp. It does so by decreasing interleukin production and immunoglobulin E (IgE), as well as reducing the expansion of regulatory T-cell populations. Hyperinfection then ensues [8].

In hyperinfection or dissemination phase, patients can present with a variety of signs and symptoms, some of which include rash, indigestion, malabsorption, cramping lower abdominal pain, intermittent or persistent diarrhea, constipation, weight loss, fever, vomiting, anal pruritus, hematemesis, gastrointestinal ulceration, perforation, or obstruction [9].

Peripheral eosinophilia may often be the first step in the pathophysiology of a parasitic infection. However, it is an inconsistent finding in chronic strongyloidiasis so that in the study by Koczka et al. [8], not all patients with documented strongyloidiasis had peripheral eosinophilia.

Strongyloidiasis is treated with three different medications, including ivermectin, albendazole, and thiabendazole [10]. The latter is not commonly used because of its severe adverse effects. Patients infected with HTLV-1, and those victims with any form of immunosuppression, tend to have lower cure rates, as was established in Koczka's study [8].

Currently, the treatment of choice for strongyloidiasis is ivermectin. Prolonged therapy with ivermectin might be needed in cases of severe immunosuppression and hyperinfection [11].

## Conclusions

This case highlights the relevance of considering Strongyloides hyperinfection as a cause of gastric outlet obstruction in the setting of immunocompromised patients who are from/or coming from the parasite endemic areas.
